# VRK1 Predicts Poor Prognosis and Promotes Bladder Cancer Growth and Metastasis *In Vitro* and *In Vivo*

**DOI:** 10.3389/fphar.2022.874235

**Published:** 2022-04-26

**Authors:** Jiacheng Wu, Tao Li, Hao Ji, Zhi Chen, Baoqian Zhai

**Affiliations:** ^1^ Department of Urology, The Affiliated Tumor Hospital of Nantong University, Nantong, China; ^2^ Department of Medical Oncology, The Affiliated Tumor Hospital of Nantong University, Nantong, China; ^3^ Department of Pathology, The First People’s Hospital of Longquanyi District, Chengdu, China; ^4^ Department of Oncology Radiotherapy, Yancheng No. 1 People’s Hospital, Yancheng, China

**Keywords:** VRK1, bladder cancer, growth and metastasis, nomogram, TCGA (the cancer genome atlas program), GEO, GSEA

## Abstract

Bladder cancer (BC) is one of the most common malignant tumors in the urinary system with growing morbidity and diagnostic rate in recent years. Therefore, identifying new molecular biomarkers that inhibit the progression of bladder cancer is needed for developing further therapeutics. This study found a new potential treatment target: vaccinia-related kinase 1 (VRK1) and explored the function and mechanism of VRK1 in the development of bladder cancer. First, TCGA database and tissue microarray analysis showed that VRK1 was significantly upregulated in bladder cancer. Kaplan–Meier survival analysis indicates that the OS and PFS of the VRK1 high expression group were significantly lower than the VRK1 low expression group (p = 0.002, p = 0.005). Cox multi-factor analysis results show that VRK1 expression is an independent risk factor affecting tumor progress. The maximum tumor diameter, staging, and adjuvant chemotherapy also have a certain impact on tumor progression (*p* < 0.05). In internal validation, the column C index is 0.841 (95% CI, 0.803–0.880). In addition, cell functional studies have shown that VRK1 can significantly inhibit the proliferation, migration, and invasiveness of bladder cancer cells. *In vivo*, nude mice transplanted tumors further prove that low VRK1 can significantly inhibit the proliferation capacity of bladder cancer cells. In summary, VRK1 expression is significantly related to the staging, grade, and poor prognosis of patients with bladder cancer. At the same time, *in vivo* and *in vitro* experiments have shown that downregulation of VRK1 can significantly inhibit the proliferation of bladder cancer cells. These findings provide a basis for using VRK1 as a potential therapeutic target for patients with bladder cancer.

## Introduction

Bladder cancer (BC) is one of the most common malignant tumors in the urinary system, and is the 11th most common cancer in the world (Torre et al., 20122015). More than 430,000 patients have been diagnosed with bladder cancer (Rajwa et al., 2018) every year, and about 165,000 people will die. Although considerable progress has been made in surgical techniques, its 5-year survival rate remains at a relatively low level (Li et al., 2017; Peng et al., 2017). Postoperative tumor recurrence and progress are the major clinical events affecting the prognosis of these patients (Wang et al., 2019). Recent research studies show that relative parameters such as postoperative pathological staging of the tumor can predict the survival and prognosis of bladder cancer patients, but the effectiveness is not reliable. Thus, new prognosis indicators and possible targets for bladder cancer are crucial.

VRK1 is a member of the vaccinia-related kinase (VRK) family of serine/threonine protein kinases. It is a Ser–Thr kinase with atypical activity (Campillo-Marcos et al., 2021), and a nucleosome kinase or chromatin kinase (Kang et al., 2007). It can interact directly and stably with different chromatin proteins, and is related to cell cycle entry, apoptosis, and autophagy control (Kim et al., 2012). It regulates a variety of transcription factors, including ATF2 (Sevilla et al., 2004), p53 (Lopez-Borges and Lazo, 2000), and c-Jun (Liu et al., 2017). All of them are highly involved in tumorigenesis. Studies have indicated that VRK1 is highly expressed in many human tumors and affects the prognosis of patients. For example, VRK1 increases the number of G1-arrested cells by reducing cyclin D1 and p-Rb while upregulating p21 and p27. Its consumption downregulates the phosphorylation of CREB to participate in the tumorigenesis and development of liver cancer (Huang et al., 2016). VRK1 relates to poor prognosis of glioma by participating in the PI3K/AKT pathway (Ben et al., 2018); studies have also shown that ginsenoside Rg3 affects DNA damage and causes VRK1 upregulation and P53BP1 foci formation, thereby inhibiting lung cancer cell viability (Liu et al., 2019). VRK1 overexpression can promote breast cancer cell mesenchymal transition (MET) by regulating the transcriptional repressor snail, slug, and twist (Mon et al., 2018). At the same time, there are related research reports on rectal adenocarcinoma (Del Puerto-Nevado et al., 2016) and other tumors. However, there is no relevant research on VRK1 and bladder cancer. Therefore, the purpose of this study is to evaluate the role of VRK1 in the prognosis of bladder cancer. In addition, our research hopes to further explore the specific biological functions of VRK1 in bladder cancer through *in vivo* and *in vitro* experiments.

## Materials and Methods

### Classification of Differentially Expressed Genes in TCGA and GEO Database

We used the GEO query package to download two RNA expression data sets GSE13507 and GSE65635 (containing tumor tissue and normal tissue) from the GEO database (https://www.ncbi.nlm.nih.gov/geo/query/acc.cgi). GEO online GEO2R tool was used to analyze the differential genes. The parameters for screening DEG are selected if the log2FC is greater than 0 and *p* value is less than 0.02. The data set of differentially expressed genes of bladder cancer is downloaded from the TCGA database (https://tcga-data.nci.nih.gov/), and the parameters for screening DEG are determined if the log2FC is greater than 0 and *p* value is less than 0.0001 (Diboun et al., 2006; Davis and Meltzer, 2007; Gu et al., 2016). Finally, the intersection of the three data sets is showed in a Venn diagram to obtain the common upregulated differentially expressed genes.

### Data Analysis Using the TCGA Database

In order to study the expression of VRK1 in bladder cancer, we used the TCGA database (https://tcga-data.nci.nih.gov/) for analysis. Among them, 414 cases of cancer tissues and 19 paracancerous tissues were included in the study. R 3.6.3 software is used to collect and analyze the data. The expression of VRK1 gene in bladder cancer tissue and paracancerous tissue was compared according to the standard calculation method as described previously.

### Gene Enrichment of VRK1

We used gene enrichment methods to further explore the possible signaling pathways VRK1 may participate in the poor prognosis of bladder cancer. GSEA analysis is mainly performed using GSEA software version 2.2.2.0 (Mootha et al., 2003), which uses a predefined gene set from the molecular signature database (MSigDB v5.0) (Subramanian et al., 2005). A gene set is a group of genes that share pathways, functions, chromosomal locations, or other characteristics. In this study, we used all the C sets for GSEA analysis (i.e., C1–C7 sets in MsigDB), and listed the ranking genes based on the score of -log10 of the *p* value multiplied by the fraction of multiple change of the expression. The gene sets collected according to the minimum and maximum selection criteria were 10 and 500 genes, respectively.

### Clinical Data Selection

The clinical data of 101 patients with bladder cancer who underwent surgical resection in the Department of Urology, Nantong Cancer Hospital from May 2013 to May 2015 were selected. Among them, 30 cases of bladder cancer were accompanied by corresponding paracancerous tissues. The inclusion criteria were: ① all patients who were diagnosed with bladder cancer for the first time and only had bladder cancer as tumor diagnosis and surgery; ② clinical staging was stage Ⅰ–Ⅲ; ③ postoperative pathology was confirmed to be bladder cancer (urothelial carcinoma); ④ have complete clinical data and follow-up data; ⑤ have no other antitumor therapy before admission (neoadjuvant chemoradiation or other antitumor therapies); and ⑥ good patient compliance. The exclusion criteria were: ① received radiotherapy, chemotherapy, hormone therapy, etc., before surgery; ② combined with other serious diseases, such as severe hypertension; ③ combined with certain acute-stage diseases, such as infection; and ④ refused to follow-up. The age range of the patients included in this study was 34–81 years old, with an average age of (63.0 ± 9.5) years. There are 82 males and 19 females. All samples were collected under the written informed consent of the participants, and the experiment was approved by the Ethics Committee of Nantong Cancer Hospital. Follow-up is carried out by outpatient review or telephone follow-up. The outpatient follow-up was conducted every 3 months in the first year after surgery, every 6 months in the second year, and once a year after 2 years until May 2020. Follow-up examination: cystoscopy, abdominal MRI, CT or color Doppler ultrasound, tumor markers, urinalysis, etc., (additional examinations might be added depending on the patient’s condition). Observation indicators: OS is defined as the time from the diagnosis of the disease to death from any cause or the cut-off time of follow-up. PFS is defined as the time from the beginning of treatment to any follow-up examination that indicates disease progression. At the end of the follow-up, if the subject was still alive but lost to the follow-up, the data from the final cut-off time will be used for statistical analysis.

### Cell Source

The five bladder cancer cell lines (J82, SW780, T24, 5637, and UM-UC-3) and a normal human bladder normal epithelial cell (SV-HUC-1) used in this study were mainly purchased from Chinese Academy of Sciences Cell bank, Shanghai.

### Tissue Microarray Construction, Immunohistochemistry, and Its Correlation With Survival Prognosis and Clinical Data Parameters

The tissue microarray was constructed by the Department of Pathology, Nantong Cancer Hospital. Hematoxylin–eosin was used to stain the paraffin embedded tissue blocks of 101 cases of bladder cancer. The most typical features were selected and marked at fixed points under the microscope. Each dot array contains less than 160 dots. A 3-micron-thick section was cut from the paraffin embedded tissue block and transferred to a glass slide using tape transfer system for UV cross-linking. IHC staining was performed according to the previously described protocol (Laurberg et al., 2014). Anti-VRK1 antibody [5D1]-N-terminal mouse monoclonal antibody (1:500, abcam ab171933) was used. The tissue sections were examined through two experienced pathologists under double-blind condition. The immunohistochemistry results were scored considering two factors: the proportion of positive cells and the intensity of cell staining. We found that VRK1 is mainly located in the nucleus. The score according to the proportion of positive staining cells: 0 (negative), 1 (<25%), 2 (25–50%), 3 (51–75%), and 4 (>75%), and staining intensity: 0 (negative or no staining), 1 (weak positive), 2 (central type), and 3 (strong positive). The value obtained by multiplying these two scores is the final score corresponding to each specimen. After calculating the arithmetic mean of these scores, the specimen with a score lower than 6 is finally defined as a low expression of VRK1. According to the expression of VRK1 under immunohistochemistry, the 101 bladder cancer patients included in the study were divided into two groups: high and low expression of VRK1. SPSS software was used to compare the correlation between the expression of VRK1 in these two groups and the survival prognosis and clinical parameters of the patients. Meanwhile, the independent prognostic factors that are meaningful for the prognosis of bladder cancer patients were further screened through uni- and multi-variate analysis.

### Reverse Transcription Quantitative PCR Method to Detect the mRNA Encoding VRK1

Take 100 mg sample of both the tumor tissue and paracancerous tissue of the patients included in the study and mill these samples into powder by the liquid nitrogen grinding method. Add 1 ml Trizol lysis solution, and extract the total RNA according to the instructions. The primers are: 5′-CTT​CAG​AGT​CAG​TTG​GCA​GTG-3′, 5′-CTT​CAG​CTT​ACG​GGT​ACG​AAT-3′; GAPDH is 5′-CAT GGG TGT GAA CCA TGA GAA GTA-3′. Use the reverse transcription kit to reverse transcribe RNA to obtain cDNA. Perform real-time fluorescent quantitative PCR on a fluorescent quantitative PCR machine. Calculate the relative expression of the target molecule’s mRNA by the 2^−△△ct^ method. Therefore, we obtained the expression of VRK1 in cancer and paracancerous tissue.

### *In vitro* Cytology Experiment Verification

#### CCK8-Detection Cell Activity

1) Screen five bladder cancer cell lines (J82, SW780, T24, 5637, and UM-UC-3) with high VRK1 expression. Construct VRK1 knockdown bladder cancer cell lines by using small interfering RNA knockdown techniques. Digest the T24 and 5637 cells transfected with these siRNA and the negative control cells separately. Centrifuge at low speed, then resuspend the target cells using complete medium.
2) Use a cell counter to adjust all sample cells to the same number (about 4*10^3^ cells in each well in a 96-well plate). Add 100 uL of culture medium per well.
3) Set up three replicate wells for each experimental group. Set complete medium as the blank control group at the same time. Shake well and then observe the cell distribution under the microscope to ensure that the cells are evenly distributed without accumulation or overlap. Transfer to 37°C, 5% CO_2_ cell incubator for overnight culture.
4) After overnight incubation, add CCK-8 reagent to each well, usually 10 ul, and then move it again into a 37°C, 5% CO_2_ cell incubator for incubation for 2 h.
5) Determine the absorbance value at 450 nm according to the instruction manual of the microplate reader. Record the OD value, and draw the proliferation curve according to the data.

#### Cloning Formation Assay

1) Prepare a suspension of the target cells. Adjust the cell density of the stock solution by cell counter. Dilute slowly till the cell density decreased to about 300 cells in each well of the 6-well plate. Cultivate for about 14 days without replacing complete medium.
2) After the incubation, absorb the original medium as much as possible. Add paraformaldehyde (4%) to fix the cells for about 20 min.
3) Rinse with pre-cooled PBS (1x) 2–3 times. After the last PBS buffer rinsing, add crystal violet staining solution (0.1%) for about 30 min to stain the cell.
4) Use pre-cooled PBS (1x) to wash 2–3 times to wash off the excess crystal violet staining solution as much as possible and calculate the number of clone groups.

#### Transwell Migration Experiment

1) Digest the selected target cells, centrifuge, and then resuspend the target cells with PBS (1x) to wash. Centrifuge and then remove the upper layer of the liquid. Resuspend the cells with serum-free medium.
2) Take the serum-free minimal medium cell stock solution for cell count. Adjust the cell density with serum-free minimal medium to reach the target cell number, about 3*10^5^.
3) Put 500 ml of complete culture (containing 10% fetal bovine serum) in the lower chamber of the sterile 24-well plate. Put the small chamber into the corresponding 24-well plate.
4) Add the cell suspension stock solution to the upper chamber of the 24-well plate; the volume is about 200 ul. Shake slightly. Incubate in cell incubator for 24 h.
5) After incubation, add about 600 ml 4% paraformaldehyde dropwise to the blank wells. Rinse the small chamber with PBS and put it back in for fixation for 20 min.
6) Generally, use PBS to rinse slowly after the fixation. After letting it air dry for a proper amount of time, stain with crystal violet staining solution (0.1%) for 30 min.
7) Gently take out the small chamber and wash it with PBS 2–3 times. Then, use a cotton swab to clean the cells that have not migrated in the upper small chamber. After drying, observe and take pictures with a microscope.

#### Transwell Invasion Experiment

##### Transwell Chamber Preparation

i) Matrigel-Free Transwell Cell Preparation
  ① Coating basement membrane: Coat the upper chamber surface of the bottom membrane of the Transwell chamber with 50 mg/L Matrigel 1:8 dilution. Air-dry at 4°C.

##### Preparation of Cell Suspension

① Before preparing the cell suspension, the cells can be de-serum–starved for 12–24 h to further remove the influence of the serum, but this step is not necessary.
② Digest the cells: centrifuge and discard the culture medium after terminating the digestion. Wash 1–2 times with PBS, and resuspend in serum-free medium containing BSA. Adjust the cell density to 1–10 × 10^5^.

##### Inoculation of the Cells

① Take 200 µl of cell suspension into the Transwell chamber.
② Generally, 500 µl of medium containing FBS is added to the lower chamber of the 24-well plate. Please refer to the instructions for details. Special attention should be paid to the fact that there are often bubbles between the lower culture medium and the chamber. Once bubbles are generated, the chemotaxis of the lower culture medium is weakened or even disappeared. Pay special attention when planting plates. Lift the chamber to remove air bubbles, and then put the chamber into the culture plate.
③ Cultivate cells: conventionally culture for 12–48 h (mainly depending on the invasiveness of the cancer cells). In addition to the invasiveness of cells, when selecting the incubation time, the influence of each process on the number of cells cannot be ignored.

##### “Adherent” Cell Count

① By staining the cells, the cells can be counted under the microscope.
② Wipe off the Matrigel and the cells in the upper chamber with a cotton swab.
③ Dyeing: The commonly used dyeing methods include crystal violet dyeing.

#### Western Blot Detection

1) Electrophoresis process: Choose a constant voltage of 80 V for the upper layer of compression gel. After the protein sample runs to the lower layer of the separation gel and the protein marker layer can be observed with the naked eye, change the voltage to 120–150 V. End the electrophoresis when the sample runs to a distance of about 0.5 cm from the bottom edge of the separation gel.
2) Transfer: Pour the 1x transfer membrane solution prepared 1 day in advance from the refrigerator into the iron pan used in the operation. Immerse the required PVDF membrane in methanol to activate (about 15–30 s) in advance, and then transfer the PVDF membrane so that it is completely immersed in ddH_2_O for depolarization. Place the PVDF membrane in the pre-cooled transfer buffer. Soak the black sponge pad and filter paper larger than the size of the PVDF membrane into the transfer fluid (1x) in advance. Gently lift one end of the short glass plate to leave the gel, and then cut out the upper layer glue and the excess part of the lower layer glue and move the gel to the black side of the transfer membrane clamp. The black side of the transfer membrane is the bottom and the white side is the top. It is placed layer by layer in the order of sponge pad, two layers of filter paper, gel, PVDF membrane, two layers of filter paper, sponge pad, etc., from bottom to top. Slowly clamp the transfer membrane and immerse into the electrophoresis tank containing the transfer buffer. It must be ensured that a suitable ice box has been placed outside the transfer tank. Add the transfer buffer to the silver wire on the upper edge of the transfer membrane clamp. Tighten the lid and put it in an ice–water mixed bath and turn on the power. Generally, the transfer uses a constant current of 300 mA for 90 min.
3) Blocking: After transfer time, immerse the PVDF membrane faceup in the pre-prepared skimmed milk blocking solution (5%), and place it in a room temperature slow-speed shaker for about 2 h.
4) Incubate the primary antibody: Prepare the wet box and the incubation tank. Dilute with the special primary antibody diluent according to the instructions of the primary antibody. Put it into the corresponding incubation tank. Wash the completely blocked PVDF membrane using TBST (1x) 2–3 times. Use filter paper to remove excess liquid. Completely soak the membrane containing the target protein facedown in the primary antibody incubation solution. Put it in the refrigerator at 4° overnight.
5) Wash the membrane: Take out the wet box from the refrigerator and let it stand for 20–30 min at room temperature. Recover the primary antibody and note the relevant information of the antibody. Put the membrane faceup in TBST (1x) to wash 3–4 times with gentle but quick shaking for cleaning. Each cleaning period should last for about 4–5 min.
6) Incubate the secondary antibody: Following the instructions of the secondary antibody, use TBST (1x) to dilute the corresponding secondary antibody to the corresponding concentration. Use filter paper to absorb the excess solution on the membrane. Turn the membrane containing the target protein facedown to ensure that it is completely immersed in the secondary antibody. Normally, it should be incubated at room temperature for about 2 h.
7) Wash the membrane again: After secondary antibody incubation, place the membrane containing the target protein faceup. Wash it in TBST (1x) 3–4 times, with gentle but quick shaking for cleaning. Each cleaning period should last for about 20 min. Soak the membrane in the last cleaning solution to prepare for Chemiluminescent.
8) Chemiluminescent: Fully mix ECL developer A and B at equal volume. Add sufficient amount of mixed liquid on the front of the membrane. Perform the chemiluminesent according to the instructions of the instrument and save the picture.

### *In Vivo* Nude Mice Experimental

For animal experiments, male NOD-SCID nude mice, 6–7 weeks old (weight 18–20 g), were purchased from the Animal Center of the Chinese Academy of Sciences. All mice are raised in the SPF animal room of the Key Laboratory of the Ministry of Health in accordance with the NIH animal experiment guidelines in the United States. In this project, we used a total of eight animals, with four animals in each group. 1) T24 bladder tumor cell lines (shVRK1-NC and shVRK1-2) at exponential growth phase were selected. Cell line was digested using Trypsin and neutralized with complete medium. Transfer it to a 50 ml sterile centrifuge tube with a pipette, centrifuge at 1000 rpm for 5 min, and then discard the supernatant. Mix the cells thoroughly with appropriate complete medium and count the cells. 2) Dilute the cells to 5 × 10^7^/ml, according to the calculated cell concentration. 3) Randomly divide the mice into an experimental group and a control group: each nude mouse is subcutaneously injected into the lower abdominal wall under the axillary region with 100 μL of 5 × 106 cells per mice with a syringe. 4) After 1 week of subcutaneous injection, measure the tumor on the abdominal wall of nude mice every 2–3 days to calculate the volume of the tumor (cm3) = width (cm) x width (cm) x length (cm)/2; 5). After 6 weeks, the nude mice of the experimental group and the control group were sacrificed using cervical dislocation. The tumors formed under the skin were collected, the size and weight were measured, and the relationship between the growth of tumor tissue and the proliferation ability of bladder cancer cells in the body were analyzed, followed by the follow-up immunohistochemistry experiment.

### Statistical Analysis

Use software R (version 3.6.3) for statistical analysis and visualization; R package: DESeq2 (version 1.26.0) (Love MI et al., 2014) for data download; limma package (version 3.42.2) for difference analysis. Use the ggplot2 package (version 3.3.3) for image visualization. Through gene set enrichment analysis (GSEA), use R package: mainly clusterProfiler package (3.14.3 version) (for GSEA analysis) to explore the possible cellular mechanism of VRK1. Use SPSS version 22.0 (IBM) software for statistical analysis. The chi-square test was used to compare and analyze the clinicopathological conditions of the two groups of patients. The Kaplan–Meier method was used to evaluate the survival of patients, and the log rank statistical method was used for significance testing. The Cox proportional hazard regression model is used to clarify the independent prognostic factors that are meaningful for the prognosis of bladder cancer patients. On this basis, the R language is used to draw a nomogram to construct a predictive model: nomogram drawing is composed of survival and rms software packages in R. The main steps for creating a nomogram model are as follows: 1) Select a research model: According to the research data and corresponding results, different models can be selected for the nomogram. This study is the survival analysis of 101 patients with bladder cancer undergoing surgical resection, so COX risk ratio model is ideal. 2) Selection of predictors: The COX risk ratio model can be used to obtain the independent risk factors and risk ratios (HR) of 101 bladder cancer patients undergoing surgical resection. R gives the impact score of each risk factor according to the degree of contribution of different risk factors to the result, and finally obtains the corresponding survival rate of the individual patient based on the total score. The difference was statistically significant with *p* < 0.05.

## Results

### Selection of Differentially Expressed Genes, Their Expression in the TCGA Database, and Using GSEA, GO, and KEGG for Gene Enrichment Analysis

**FIGURE 1:**
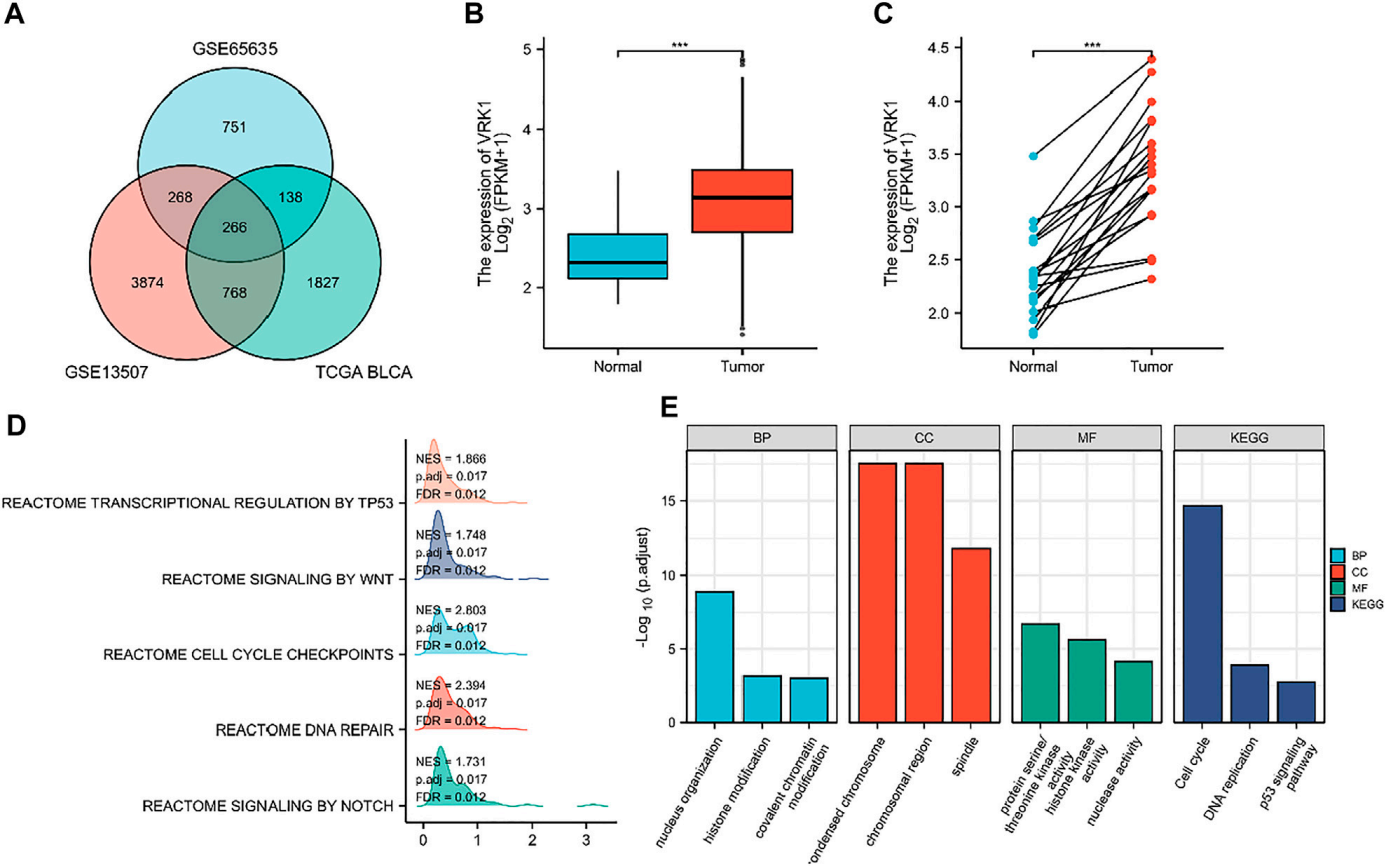
Differentially expressed genes (DEGs) and their expression in the TCGA database and gene enrichment analysis results of VRK1. **(A)** Venn diagrams of upregulated genes in three data sets. **(B)** VRK1 in unmatched bladder cancer tissues (n = 414) and paracancerous tissues (n = 19) in the TCGA database is highly expressed in tumor tissues. **(C)** VRK1 in the matched bladder cancer tissues (n = 19) and paracancerous tissues (n = 19) in the TCGA database is highly expressed in tumor tissues. **(D)** GSEA analysis results for VRK1. **(E)** Functional analysis results of GO and KEGG. Note: ***<0.001, the difference is statistically significant.

We used the GEO query package to download two RNA expression data sets GSE13507 and GSE65635 (containing tumor tissue and normal tissue) from the GEO database (https://www.ncbi.nlm.nih.gov/geo/query/acc.cgi). We used the GEO online GEO2R tool to analyze the differential genes. The parameters for screening DEG were selected if the log2FC is greater than 0 and *p* value is less than 0.02. Then, the data set of differentially expressed genes for bladder cancer from the TCGA database was downloaded (https://tcga-data.nci.nih.gov/). The parameters for screening DEG were selected if the log2FC is greater than 0 and *p* value is less than 0.0001 (Figure 1A). The differentially expressed gene is screened and confirmed to be VRK1. In order to confirm the expression level of VRK1 in bladder cancer tissues, we used the TCGA database (https://tcga-data.nci.nih.gov/) to search and analyze the mRNA expression level of VRK1. Meanwhile, we found that compared with the paracancerous tissues in the TCGA database, the mRNA expression of VRK1 in tumor tissues is upregulated (Figures 1B,C).

In order to further explore the possible role of the differentially expressed genes screened in bladder cancer, we first predicted the possible related molecular mechanisms through GSEA. We found that VRK1 may participate in DNA repair, cell cycle checkpoints, NOTCH signaling pathways, WNT signal pathway, and the transcription and regulation of TP53 to affect the biological process of bladder cancer, leading to different prognosis of bladder cancer (Figure 1D). We also used Metascape for online functional analysis. The differential genes were added to Metascape for functional analysis of GO and KEGG. GO-BP analysis showed that nucleus organization, histone modification, and covalent chromatin modification are possible cytological behaviors involved. GO-CC analysis found that condensed chromosome, chromosomal region, and spindle are possible sites involved. GO-MF analysis found that protein serine/threonine kinase activity, histone kinase activity, and nuclease activity are the molecular functions that may be affected. KEGG analysis found that cell cycle, DNA replication, and p53 signaling pathway are signaling pathways that may be affected (Figure 1E).

### Expression of VRK1 in Bladder Cancer Tissue and VRK1 Knockdown can Significantly Inhibit the Proliferation and Clonal Formation Assay of Bladder Cancer Cells

**FIGURE 2:**
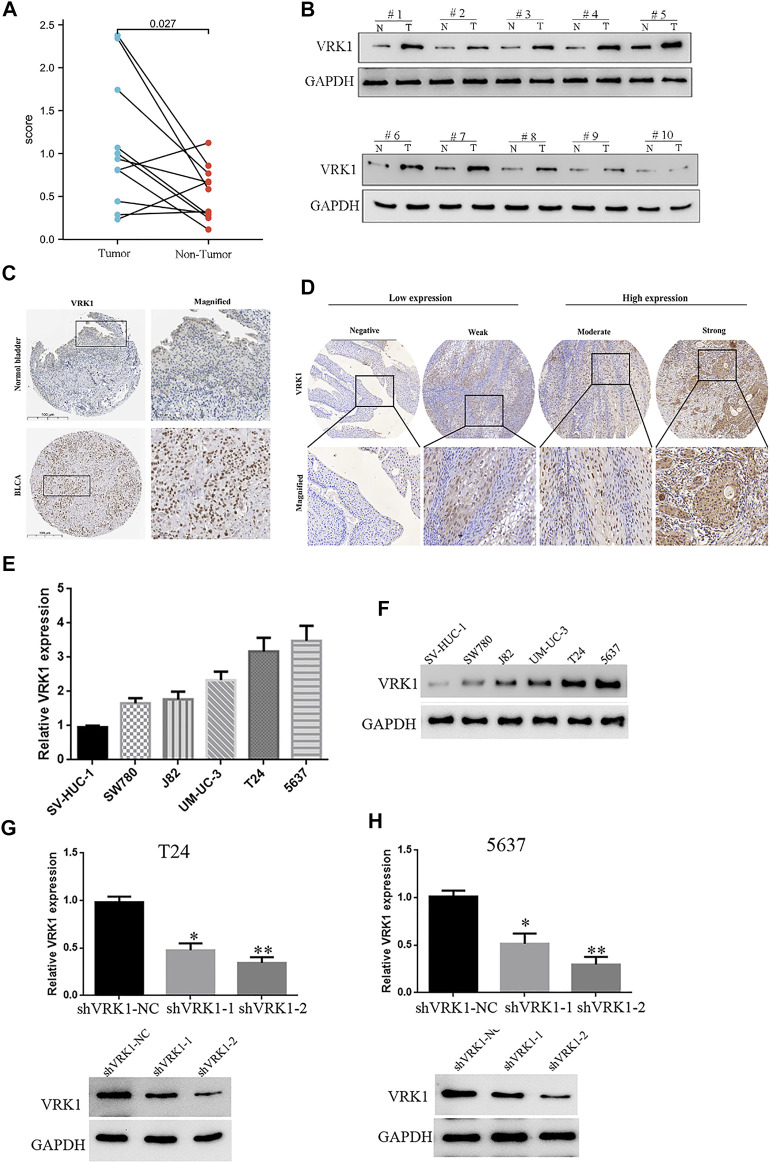
High expression of VRK1 in bladder cancer tissues and bladder cancer cell lines. **(A)** Reverse transcription quantitative PCR showed that the expression of VRK1 in cancer tissues was higher than that in paracancerous tissues at the mRNA level (*p* < 0.05). **(B)** Western blot experiments showed that the expression of VRK1 in cancer tissues was higher than that in paracancerous tissues at the protein level (*p* < 0.05). **(C)** In The Human Protein Atlas database, VRK1 is higher expressed in cancerous tissues than in paracancerous tissues, and is mainly expressed in the nucleus. **(D)** Immunohistochemistry showed that VRK1 was mainly expressed in the nucleus. **(E)** Fluorescence qPCR to detect the expression of VRK1 in bladder cancer cell lines and normal human bladder normal epithelial cells. The experiments were repeated three times. **(F)** Western blot to detect the expression of VRK1 in bladder cancer cell lines and normal human bladder normal epithelial cells. **(G,H)** Fluorescence qPCR and Western blot to detect the expression in bladder cancer T24 and 5637 cells after knocking down VRK1, *p < 0.05, **p < 0.01.

The tumor tissues and paracancerous tissues of the patients included in the study were taken to compare the VRK1 expression. Using reverse transcription quantitative PCR (Figure 2A) and Western blotting (Figure 2B), we found that VRK1 expression in cancer tissues is higher than that in adjacent tissues (*p* < 0.05). The Human Protein Atlas (https://www.proteinatlas.org/) database indicates that VRK1 has a higher expression in cancerous tissues than in paracancerous tissues. Moreover, the gene is mainly located in the nucleus, with a small amount expressed in the cytoplasm (Figure 2C). We used immunohistochemistry to check the expression of VRK1 in the pathological tissues studied in this project. We found that VRK1 is also mainly expressed in the nucleus (Figure 2D), which is consistent with the expression in the database. Moreover, VRK1 was low expressed in 43 bladder cancer tissue samples (42.6%), and highly expressed in 58 bladder cancer tissue samples (57.4%).

The aforementioned results indicate that VRK1 is highly expressed in bladder cancer tissues. Therefore, in order to study the specific biological function of this gene in bladder cancer, it is planned to use bladder cancer cell lines for further functional research. qRT-PCR was used to detect the expression level of VRK1 in 5 bladder cancer cell lines (J82, SW780, T24, 5637, and UM-UC-3) and a normal human bladder normal epithelial cell (SV-HUC-1). The results of fluorescence qPCR and Western blot showed that the expression levels of VRK1 in various bladder cancer cell lines are quite different, with the highest expression levels in T24 and 5637 cells, followed by UM-UC-3, J82, and SW780 cells (Figures 2E,F). Therefore, in the follow-up cell function research, we selected VRK1 highly expressing T24 and 5637 cells as the research objects to conduct in-depth cell function and molecular mechanism research. First, we constructed two lentiviral-mediated RNA interference vectors targeting VRK1 gene (shVRK1-1 and shVRK1-2) and shVRK1-control (shVRK1-NC) in T24 and 5637 cells, respectively, and then detected knockdown by fluorescent qPCR and Western blot. The results suggested: compared with the blank control group (shVRK1-NC), shVRK1-1 and shVRK1-2 can significantly knockdown the expression level of VRK1 in bladder cancer cells after transfection, and shVRK1-2 has a higher knockdown efficiency (Figures 2G,H). The aforementioned results indicate that the bladder cancer cell line with VRK1 knockdown is successfully constructed, providing experimental materials for subsequent functional studies.

### Survival and Prognosis of Patients Included in This Study

**FIGURE 3:**
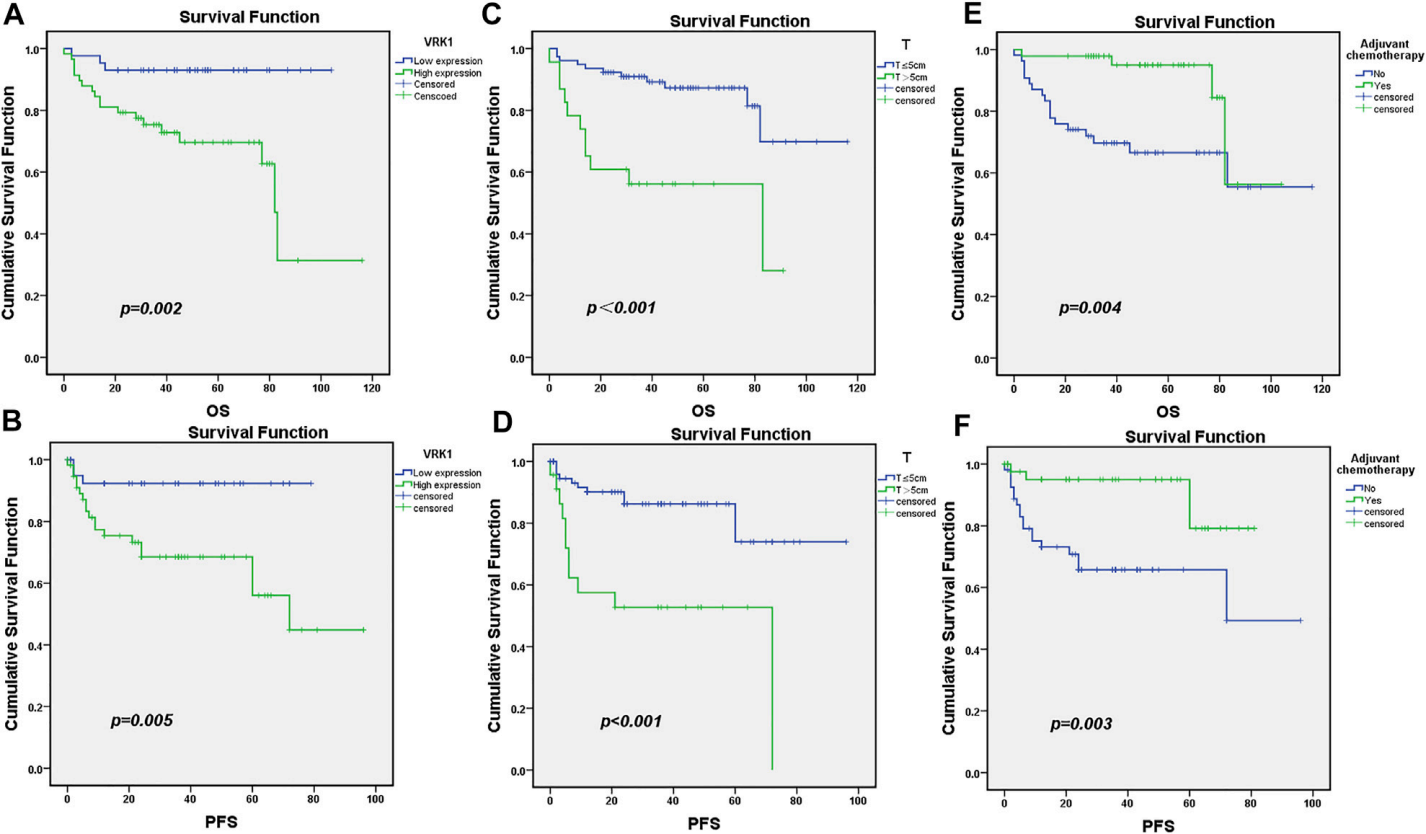
Survival and prognosis of patients included in the study. **(A,B)**: OS and PFS of patients in the VRK1 low expression group are better than those in the VRK1 high expression group. **(C,D)**: OS and PFS of patients with tumor diameter ≤5 cm are better than those in the group with tumor diameter >5 cm. **(E,F)**: OS and PFS of patients that have undergone adjuvant chemotherapy are better than those of those who have not undergone adjuvant chemotherapy.

All patients were followed up for 5 years. A total of 79 patients survived, with a survival rate of 78.2%, and 22 patients died, with a mortality rate of 21.8%. Kaplan–Meier survival analysis results showed that the OS and PFS of patients in the VRK1 high expression group were significantly lower than those in the VRK1 low expression group (p = 0.002, p = 0.005) (Figures 3A,B). Meanwhile, further stratified analysis also reveals that patients with tumor diameters ≤5 cm and undergoing adjuvant chemotherapy had better OS and PFS (Figures 3C–F).

### Relationship Between VRK1 and Clinicopathological Data of Patients With Bladder Cancer

Among the 101 bladder cancer patients included, 43 cases (42.6%) were in the VRK1 low expression group, and 58 cases (57.4%) were in the VRK1 high expression group. The difference between two groups of patients in terms of gender, age, recurrence, surgical methods, adjuvant chemotherapy, invasion of the bladder triangle, tumor invasion, vascular tumor thrombus, T stage, grade, lymph node metastasis, and presence or absence of distant metastasis was not statistically significant (*p* > 0.05), but the difference in tumor size was statistically significant (*p* < 0.05) (Table 1). This also provides some reference for our follow-up experiments to explore the role of VRK1 in the tumorigenesis and development of bladder cancer.

**TABLE 1:** Relationship between the expression of VRK1 and clinicopathological data.

Variable	Total	Low expression	High expression	p
	n (%)	n (%)	n (%)	
Gender				
Male	82 (81.2)	37 (36.6)	45 (44.6)	0.282
Female	19 (18.8)	6 (5.9)	13 (12.9)	
Age				
≤65	56 (55.4)	21 (20.8)	35 (34.7)	0.250
>65	45 (44.6)	22 (21.8)	23 (22.8)	
Recurrence				
Yes	23 (22.8)	11 (10.9)	12 (11.9)	0.562
No	78 (77.2)	32 (31.7)	46 (45.5)	
Tumor size (cm)				
≤5	78 (77.2)	38 (37.6)	40 (39.6)	0.021^a^
>5	23 (22.8)	5 (5.0)	18 (17.8)	
Operation mode				
Transurethral resection	50 (49.5)	23 (22.8)	27 (26.7)	0.491
Radical cystectomy	51 (50.5)	20 (19.8)	31 (30.7)	
Adjuvant chemotherapy				
Yes	47 (46.5)	24 (23.8)	23 (22.8)	0.107
No	54 (53.5)	19 (18.8)	35 (34.7)	
Bladder triangle involvement				
Yes	36 (35.6)	15 (14.9)	21 (20.8)	0.891
No	65 (64.4)	28 (27.7)	37 (36.6)	
External invasion				
Yes	13 (12.9)	6 (5.9)	78 (6.9)	0.780
No	88 (87.1)	37 (36.6)	51 (50.5)	
Vascular tumor thrombus				
Yes	2 (2.0)	0 (0.0)	2 (2.0)	0.219
No	99 (98.0)	43 (42.6)	56 (55.4)	
T stage				
T1	22 (21.8)	9 (8.9)	13 (12.9)	0.858
T2+T3	79 (78.2)	34 (33.7)	45 (44.6)	
Grade				
G1+G2	53 (52.5)	26 (25.7)	27 (26.7)	0.166
G3	48 (47.5)	17 (16.8)	31 (30.7)	
Lymph node metastasis				
Yes	32 (31.7)	14 (13.9)	18 (17.8)	0.871
No	69 (68.3)	29 (28.7)	40 (39.6)	
Other site metastasis				
Yes	10 (9.9)	3 (3.0)	7 (6.9)	0.397
No	91 (90.1)	40 (39.6)	51 (50.5)	

a <0.05, the difference is statistically significant.

### Results of Univariate Analysis and Multi-Factor Analysis

In order to further determine the risk factors associated with OS in patients with bladder cancer, we conducted univariate and multivariate analysis to confirm whether VRK1 is an independent risk factor for poor prognosis. Univariate analysis showed that the maximum pathological diameter of the tumor, whether adjuvant chemotherapy, the presence or absence of vascular tumor thrombus, T stage, and VRK1 expression are factors that affect the patient’s OS (*p* < 0.05). The results of Cox multivariate analysis showed that the expression of VRK1 is an independent risk factor affecting tumor progression. The pathological maximum diameter, staging, and adjuvant chemotherapy also have a certain impact on tumor progression (*p* < 0.05) (Table 2).

**TABLE 2:** Univariate analysis and multivariate analysis of clinical factors on 101 patients with OS.

Variable	Univariate analysis			Multivariate analysis		
	HR	(95%CI)	p	HR	(95%CI)	P
Gender						
Male	1.019	(0.344–3.021)	0.972			
Female	1					
Age						
≤65	1	(0.953–5.427)	0.064			
>65	2.274					
Recurrence						
Yes	0.896	(0.332–2.417)	0.829			
No	1					
Tumor size (cm)						
≤5	1	(1.834–9.875)	0.001^a^	1	(1.182–6.719)	0.019^a^
>5	4.256			2.818		
Operation mode						
Transurethral resection	1	(0.704–4.075)	0.240			
Radical cystectomy	1.693					
Adjuvant chemotherapy						
Yes	0.230	(0.078–0.684)	0.008^a^	0.275	(0.088–0.858)	0.026^a^
No	1			1		
Bladder triangle involvement						
Yes	2.271	(0.965–5.347)	0.060			
No	1					
External invasion						
Yes	1.469	(0.493–4.374)	0.490			
No	1					
Vascular tumor thrombus						
Yes	11.994	(2.633–54.642)	0.001^a^			
No	1					
T stage						
T1	1	(1.164–78.517)	0.036^a^	1	(1.241–147.896)	0.033^a^
T2+T3	9.561			13.547		
Grade						
G1+G2	1	(0.792–4.527)	0.151			
G3	1.893					
Lymph node metastasis						
Yes	0.949	(0.383–2.347)	0.909			
No	1					
Other site metastasis						
Yes	2.319	(0.779–6.897)	0.131			
No	1					
Expression of VRK1						
Low expression	1	(1.672–19.238)	0.005^a^	1	(1.114–13.338)	0.033^a^
High expression	5.671			3.854		

Note: ^a^<0.05, the difference is statistically significant.

### Nomogram Construction

**FIGURE 4:**
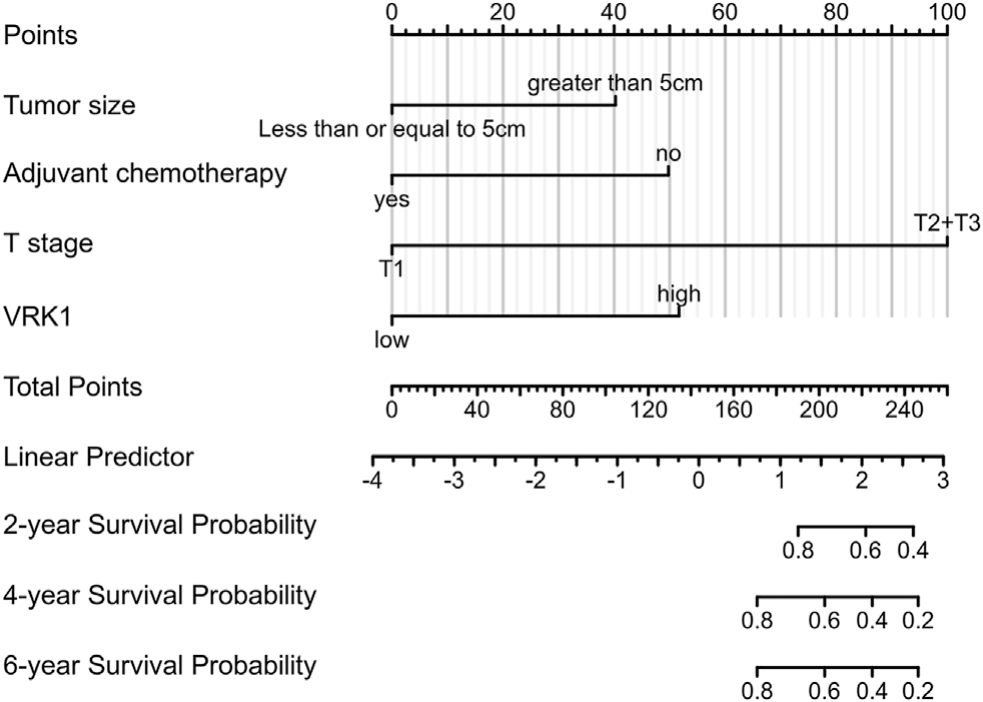
Nomogram predicts the overall survival of bladder cancer patients. For each patient, draw four lines up to determine the points received from the four predictors in the nomogram. The sum of these points is on the “Total Points” axis. Then, draw a line down to determine the probability of 2-, 4-, and 6-year overall survival for bladder cancer. * The C-index is 0.841 (95% CI is 0.803–0.880).

Predicting the 2-, 4-, and 6-year survival rates of patients with bladder cancer has a certain guiding effect to improve the prognosis of patients with poor prognostic characteristics in the clinical practice. Compared with the nomogram constructed using a single prognostic factor, the nomogram constructed by a combined model might be the bettter nomogram for predicting short-term survival rates (2, 4, and 6 years). We constructed a nomogram based on Cox regression analysis. The model successfully included 4 independent prognostic factors, including tumor diameter, adjuvant chemotherapy, T stage, and VRK1 expression. Predictive models were contracted with internal validation. In internal validation, the nomogram C-index was 0.841 (95% CI, 0.803–0.880) (Figure 4). Therefore, the prediction model has better accuracy.

### Knockdown of VRK1 Significantly Inhibits the Invasion and Migration of Bladder Cancer Cells

**FIGURE 5:**
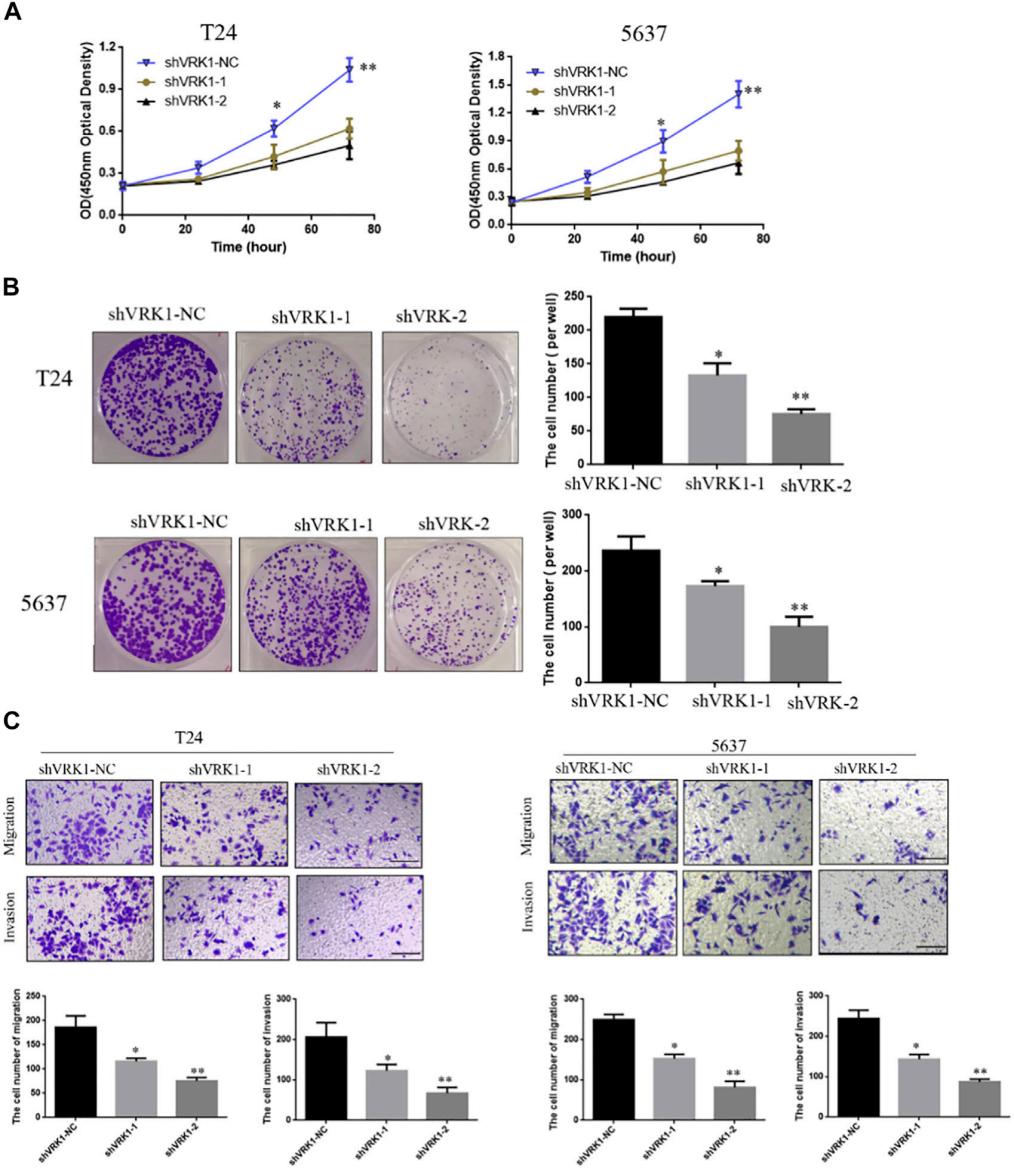
Knockout of VRK1 inhibited the proliferation, clonal capacity, migration, and invasion of bladder cancer T24 and 5637 cells. **(A)** CCK-8 detects the effect of knocking down VRK1 on the proliferation of bladder cancer T24 and 5637 cells. **(B)** Clone formation assay: The effect of knocking down VRK1 on the cloning ability of bladder cancer T24 and 5637 cells. **(C)** The effect of knocking down VRK1 on the migration and invasion of bladder cancer T24 and 5637 cells. *p < 0.05, **p < 0.01; the scale is 100 μm.

The results showed that compared with the blank control group (shVRK1-NC), shVRK1-1 and shVRK1-2 began to significantly inhibit cell proliferation at 48 h. The inhibition phenomenon is particularly significant when cultured to 72 h (Figure 5A). The results of the clone formation assay showed that compared with the blank control group (shVRK1-NC), shVRK1-1 and shVRK1-2 could significantly inhibit the number of T24 and 5637 clones (Figure 5B). This indicates that knocking down VRK1 can significantly inhibit the proliferation of bladder cells (T24 and 5637).

In order to further study the role of VRK1 in the invasion and migration of bladder cancer cells, Transwell was used to detect the invasion and migration ability of T24 and 5637 cells after knocking down VRK1. The test results showed that compared with the blank control group (shVRK1-NC), knocking down VRK1 (shVRK1-1 and shVRK1-2) can significantly inhibit the invasion and migration of T24 and 5637 cells (Figure 5C).

### Knockdown of VRK1 Significantly Inhibits the Expression of Proliferation- and Invasion-Related Molecules in Bladder Cancer Cells

**FIGURE 6:**
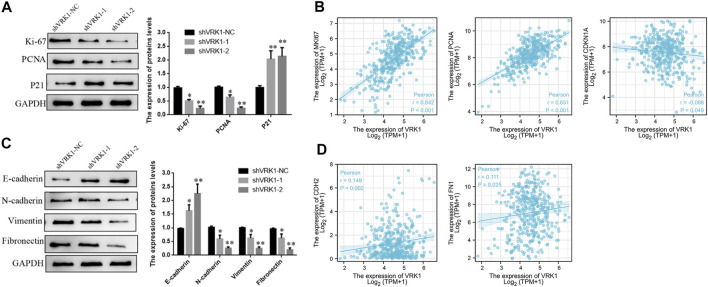
Knockdown of VRK1 inhibited proliferation and invasion in BLCA cells. **(A)** A significant reduction of Ki-67 and PCNA but increase P21 was detected in shVRK1 cells compared to shNC cells by Western blotting. GAPDH was used as an internal control. Data are presented as means ± SD of three independent experiments. **(B)** The correlation between VRK1 and Ki67, PCNA, P21 (CDNK1A) at the mRNA level was detected in the TCGA database **(C)** A significant reduction of N-cadherin, vimentin and Fibronectin but increase E-cadherin was detected in shVRK1 cells compared to shNC cells by Western blotting. GAPDH was used as an internal control. Data are presented as means ± SD of three independent experiments. **(B,D)** The correlation between VRK1 and N-cadherin (CDH2) and Fibronectin (FN1) at the mRNA level was detected in the TCGA database *p < 0.05, **p < 0.01.

The aforementioned results indicate that knockdown of VRK1 can significantly inhibit the proliferation and invasion of bladder cancer cells. To further detect whether knockdown of VRK1 affects the proliferation- (Ki-67, PCNA, and p21) and invasion- (E-cadherin, N-cadherin, vimentin, and fibronectin) related molecules of bladder cancer T24 cells, Western blot was performed. Results suggested that compared with the blank control group (shVRK1-NC), knocking down VRK1 can significantly affect the expression of proliferating cell nuclear antigen Ki-67 and PCNA, and promote the expression of cell cycle inhibitory protein p21 (Figure 6A). In addition, knocking down VRK1 can significantly promote the expression of E-cadherin and inhibit the expression of N-cadherin, vimentin, and fibronectin (Figure 6C). The abovementioned results indicate that knocking down VRK1 can significantly inhibit the expression of proliferation and invasion-related molecules in T24 cells. Moreover, we found that VRK1 gene was positively correlated with Ki-67 (MKI67), PCNA, N-cadherin (CDH2), and Fibronectin (FN1), and negatively correlated with P21(CDKN1A) in TCGA database (Figures 6B,D).

### Knockdown of VRK1 Significantly Inhibits the Proliferation of Bladder Cancer Cells *in vivo*

**FIGURE 7:**
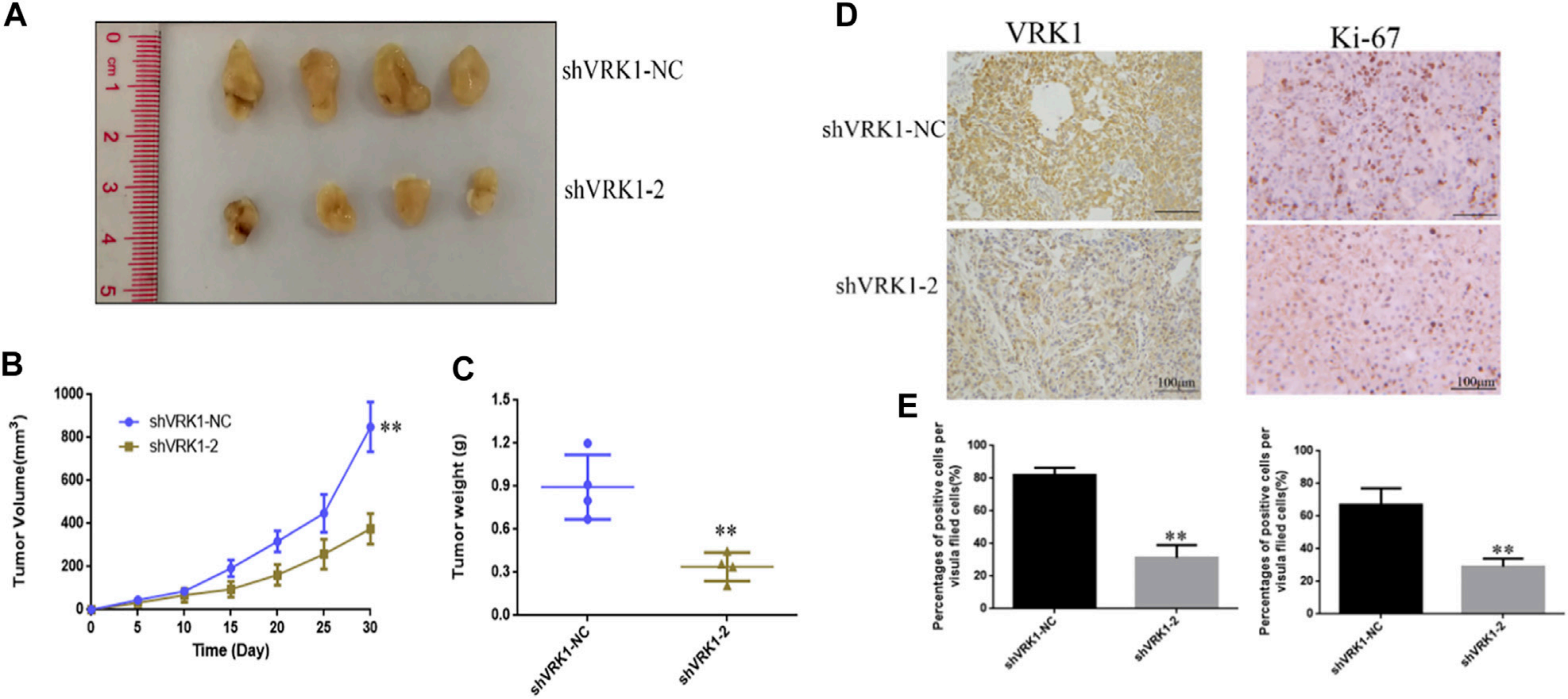
Knockdown of VRK1 significantly inhibits the proliferation of bladder cancer cells *in vivo*. **(A)** Pictures after stripping the tumor-bearing tissue from nude mice. **(B)** Measure the tumor-bearing tissue every 5 days. **(C)** Weigh the tumor-bearing tissue. **(D,E)** Immunohistochemistry to detect the expression of VRK1 and Ki-67 in tumor-bearing tissues. *p < 0.05, **p < 0.01.

In order to further study the effect of knocking down VRK1 on the proliferation of bladder cancer cells *in vivo*, the shVRK1-NC and shVRK1-2 bladder cancer T24 cells transfected with lentivirus were collected, and then injected into nude mice under the skin. The growth of tumor-bearing tissues was observed and recorded. After 30 days, the nude mice were sacrificed, and the tumor-bearing tissue was taken out for photographing and measurement. The results manifest that compared with the blank control group (shVRK1-NC), knocking down VRK1-2 can significantly inhibit the growth of bladder cancer cell T24 *in vivo* (Figures 7A,B). Then, the stripped tumor-bearing tissue was weighed, and the results imply that compared with the blank control group (shVRK1-NC), the proliferation ability of bladder cancer cell T24 *in vivo* was significantly reduced after knocking down VRK1-2 (Figure 7C). The aforementioned results show that knocking down VRK1 significantly inhibits the proliferation of bladder cancer cells *in vivo*.

In order to further detect the expression of VRK1 and proliferation-related proteins in tumor-bearing tissues, after fixing the tumor-bearing tissues, immunohistochemistry was used to detect the expression of VRK1 and Ki-67 proteins in the tumor-bearing tissues. The results suggest, compared with the blank control group (shVRK1-NC) tumor-bearing tissue, the expression of VRK1 in the tumor-bearing tissue of the knockdown group (shVRK1-2) was significantly reduced. Similarly, compared with the blank control group (shVRK1-NC) tumor-bearing tissue, the expression level of Ki-67 in the knockdown group (shVRK1-2) tumor-bearing tissue was significantly reduced (Figures 7D,E).

## Discussion

In this study, we first screened the differential genes of bladder cancer from GEO and TCGA data, and screened the differentially expressed molecules VRK1 from the differential genes. Further data analysis found that the expression differences of VRK1 is not only in between the cancer and paracancerous tissues as described in the database but also in the clinical data we included, which related to the prognosis of patients. The expression level of VRK is an independent risk factor for the prognosis of bladder cancer. We found that patients with low expression of the VRK1 molecule have a better prognosis. Then, we verified from the cytology and the nude mouse level that the reduction of VRK1 expression can inhibit the proliferation and invasion of bladder cancer cells. These results also consistently reflect the potential importance of using VRK1 to assess the prognosis of bladder cancer.

At the beginning of this study, we downloaded two RNA expression data sets GSE13507 and GSE65635 (containing tumor tissue and normal tissue) through GEO database (https://www.ncbi.nlm.nih.gov/geo/query/acc.cgi) *via* GEO query pack. The GEO online GEO2R tool was used to analyze the differential genes, using log2FC greater than 0 and *p* value less than 0.02 as the parameters to determine the screening DEG. Then, the data set of differentially expressed genes for bladder cancer was downloaded from the TCGA database (https://tcga-data.nci.nih.gov/), and the parameters for screening DEG according to log_2_FC greater than 0 and *p* value less than 0.0001 were determined. Finally, the intersection of the three data sets is mapped in a Venn diagram to obtain the common upregulated differentially expressed genes. Thus, we determined our research molecule VRK1, and verified in the TCGA database that the expression of VRK1 in cancer tissues is higher than that in paracancerous tissues. The current clinical treatment of bladder cancer is now in the era of immunotherapy, and previous analysis has shown that tumor infiltrating lymphocytes are an independent predictor of the sentinel lymph node status and survival rate in cancer patients (Azimi et al., 2012). We also used the established computing resource (CIBERSORT) (http://cibersort.stanford.edu/) to explore the gene expression profile of the downloaded sample (Gentles et al., 2015; Ali et al., 2016; Bense et al., 2017). A total of 24 types of TIIC immune response in bladder cancer are detected to evaluate their correlation with survival and molecular subgroups. We tried to find out whether the expression of VRK1 is related to the immune infiltration of bladder cancer. We were surprised to find that pDC, NK cells, Th2 cells, Tgd, T-helper cells, NK CD56 bright cells, and mast cells are the main immune cells affected by the expression of VRK1 (*p* < 0.001). Therefore, the expression of VRK1 may also have a certain relationship with the immune infiltration of bladder cancer (Supplementary Figure S1). We used the string database to analyze related genes that interact with VRK1 from the perspectives of protein–protein, protein–DNA and genetic interactions, pathways, physiological and biochemical reactions, gene and protein expression, protein domains, and phenotype screening to construct a protein–protein interaction (PPI) network. Based on the strength of the interaction, we selected the top 100 genes from this database (Supplementary Figure S2). We also used GSEA to predict possible related molecular mechanisms. We found that VRK1 may affect the biological processes of bladder cancer by participating in DNA repair, cell cycle checkpoints, NOTCH signaling pathway, WNT signaling pathway, and transcription and regulation of TP53, eventually leading to different prognosis of bladder cancer. We also used Metascape for online functional analysis. The differential genes were added to Metascape for functional analysis of GO and KEGG. GO-BP analysis found that nucleus organization, histone modification, and covalent chromatin modification are possible cytological behaviors involved. GO-CC analysis found that condensed chromosome, chromosomal region, and spindle are possible sites involved. GO-MF analysis found that protein serine/threonine kinase activity, histone kinase activity, and nuclease activity are the molecular functions that may be affected. KEGG analysis found that cell cycle, DNA replication, and p53 signaling pathway may be affected. Studies have found that VRK1 has regulatory effects on transcription factors such as p53 (Yokobori et al., 2020), ATF2 (Sevilla et al., 2004), CREB (Kim et al., 2014), c-Jun (Martín-Doncel et al., 2019), and p300 acetyltransferase (Valbuena et al., 2008). Moreover, these transcription factors have a regulatory effect on both the progression and evolution of tumors.

Studies have also pointed out that VRK1 promotes cisplatin resistance by upregulating c-MYC through c-Jun activation and serves as a therapeutic target for esophageal squamous cell carcinoma (Liu et al., 2017). The mutual influence of VRK1 and NOTCH pathways is related to the prognosis of laryngeal cancer (de Miguel-Luken et al., 2016). VRK1 may provide new potential biomarkers for improving the prognosis and treatment of Wilms tumor (WT) patients by showing the WNT signaling pathway (Liu et al., 2021). At the same time, the WNT pathway is also a crucial pathway for regulating epithelial mesenchymal EMT. VRK1 is a member of the VRK family, which also includes VRK2 and VRK3. These members have also been reported to be mostly related to the poor prognosis of tumors. For example, VRK1 is highly expressed in many human tumors and affects the prognosis of patients. For example, the consumption of VRK1 can delay cell cycle progression and reduce the proliferation of liver cancer cells, that is, the high expression of VRK1 contributes to the cell proliferation and survival of hepatocellular carcinoma (Huang et al., 2016). VRK2 regulates tumor cell invasion through the over-activation of NFAT1 and the expression of cyclooxygenase 2 (Vázquez-Cedeira and Lazo, 2012). VRK3 is involved in the cell cycle regulation, DNA repair, and neuronal differentiation of diffuse pontine gliomas, which are the essential genes for tumor cell survival (Silva-Evangelista et al., 2019). These mature research studies also provide better help for follow-up research on the role of VRK1 in bladder cancer.

The key factors in predicting outcomes and discovering the biological mechanisms leading to poor prognosis are two important parts in cancer research (Quan et al., 2015). We verified the bladder cancer tissue of the patients we included in the study by reverse transcription qPCR, and found that the expression of VRK1 in cancer tissues was significantly higher than that in paracancerous tissues. The immunohistochemistry results also confirmed that VRK1 is highly expressed in bladder cancer tissues and mainly expressed in the nucleus. These results are also consistent with the expression of VRK1 in the database. We further analyzed the expression of VRK1 with the patient’s prognosis and clinical parameters. The results reveal that the OS and PFS of patients in the VRK1 high expression group were significantly lower than those in the VRK1 low expression group (*p* < 0.05), which was also comparable to the immunohistochemistry results that patients with high VRK1 expression had worse tumor differentiation than patients with low VRK1 expression. In the analysis of clinical data, among 101 bladder cancer patients included, 43 cases (42.6%) were in the VRK1 low expression group, and 58 cases (57.4%) were in the VRK1 high expression group. The difference in tumor size between the two groups of patients was statistically significant (*p* < 0.05). This also provides some reference for our follow-up experiments to explore the role of VRK1 in the biological process of the tumorigenesis and development of bladder cancer. Further COX regression analysis indicated that the expression of VRK1 is an independent risk factor affecting tumor progression. The pathological maximum diameter, staging, and adjuvant chemotherapy also have a certain impact on tumor progression (*p* < 0.05). Based on the results of Cox regression analysis, we constructed a nomogram through a combined model and conducted internal verification, so as to have a more accurate judgment on the prediction of the 2-, 4-, and 6-year survival rate of bladder cancer patients. This is useful for the development of clinical practice. In internal verification, the C-index of the nomogram is 0.841 (95% CI is 0.803–0.880), so this prediction model also has good accuracy. Our analysis of health information and clinical data have reflected that the high expression of VRK1 is related to the poor prognosis of patients with bladder cancer, which has also aroused our interest in further research and exploration at the level of cytology and nude mice.

At the cytological level, we first used qRT-PCR to detect the expression level of VRK1 in five bladder cancer cell lines (J82, SW780, T24, 5637, and UM-UC-3) and a normal human bladder normal epithelial cell (SV-HUC- 1). The T24 and 5637 cells with high expression of VRK1 were selected as the research objects through fluorescence qPCR and Western blot. We constructed VRK knockdown cell line through lentivirus-mediated RNA interference vector for subsequent functional studies. Both CCK-8 and clone formation assay showed that knocking down VRK1 can significantly inhibit the proliferation of bladder cells (T24 and 5637). Transwell experiment results show that knocking down VRK1 can also significantly inhibit the invasion and migration ability of T24 and 5637 cells. To our surprise, further experimental studies found that knocking down VRK1 can significantly affect the expression of proliferating cell nuclear antigen Ki-67 and PNCA and promote the expression of cell cycle inhibitory protein p21. This is also consistent with the study reported by Huang et al. (2016) that the low expression of VRK1 in hepatocellular carcinoma can stabilize p53, thereby promoting the expression of P21, causing necessary cell cycle arrest and leading to a better prognosis. Knockdown of VRK1 can significantly promote the expression of E-cadherin and inhibit the expression of N-cadherin, vimentin, and fibronectin. This is also consistent with the abovementioned prediction and analysis results of GSEA that VRK1 may participate in the proliferation and metastasis of bladder cancer through certain pathways. The results of *in vitro* cytology experiments all show that VRK1 exhibits a kind of “oncogene” performance in bladder cancer cell lines. In order to better study the possible mechanism of VRK1 in the biology of bladder cancer, we conducted *in vivo* experiments. We collected shVRK1-NC and shVRK1-2 T24 bladder cancer cells transfected with lentivirus, and then injected them under the skin of nude mice to observe and record the growth of tumor-bearing tissues. By comparing the weight of tumor-bearing tissues, we found that VRK1 knockdown can significantly inhibit the proliferation of bladder cancer cells in the body. Further immunohistochemistry results of tumor-bearing tissues suggested that the expression of VRK1 in tumor-bearing tissues of the knockdown group (shVRK1-2) was significantly lower than that of the blank control group (shVRK1-NC). Compared with the blank control group (shVRK1-NC), the expression level of Ki-67 in the knockdown group (shVRK1-2) tumor-bearing tissue was significantly reduced. *In vivo* experimental results also indicate that VRK1 has the expression status of “oncogenes” in the bladder cancer tissue.

## Conclusion

Our research results range from bioinformatics data analysis to patient clinical data comparison and from *in vitro* cytology experiments to *in vivo* nude mouse experiments. All research results consistently reflect the positive relationship between low VRK1 expression and better prognosis of bladder cancer. Therefore, further research is necessary to clarify more specific molecular mechanisms, which will also be helpful for the development of new drug candidates for targeted therapies for bladder cancer.

## Data Availability

The datasets presented in this study can be found in online repositories. The names of the repository/repositories and accession number(s) can be found in the article/Supplementary Material.
