# Supplementary figures and images for: VRK1 Predicts Poor Prognosis and Promotes Bladder Cancer Growth and Metastasis In Vitro and In Vivo
Source: Front Pharmacol. 2022 Apr 26;13:874235. doi: 10.3389/fphar.2022.874235 (PMC9086458; doi:10.3389/fphar.2022.874235)

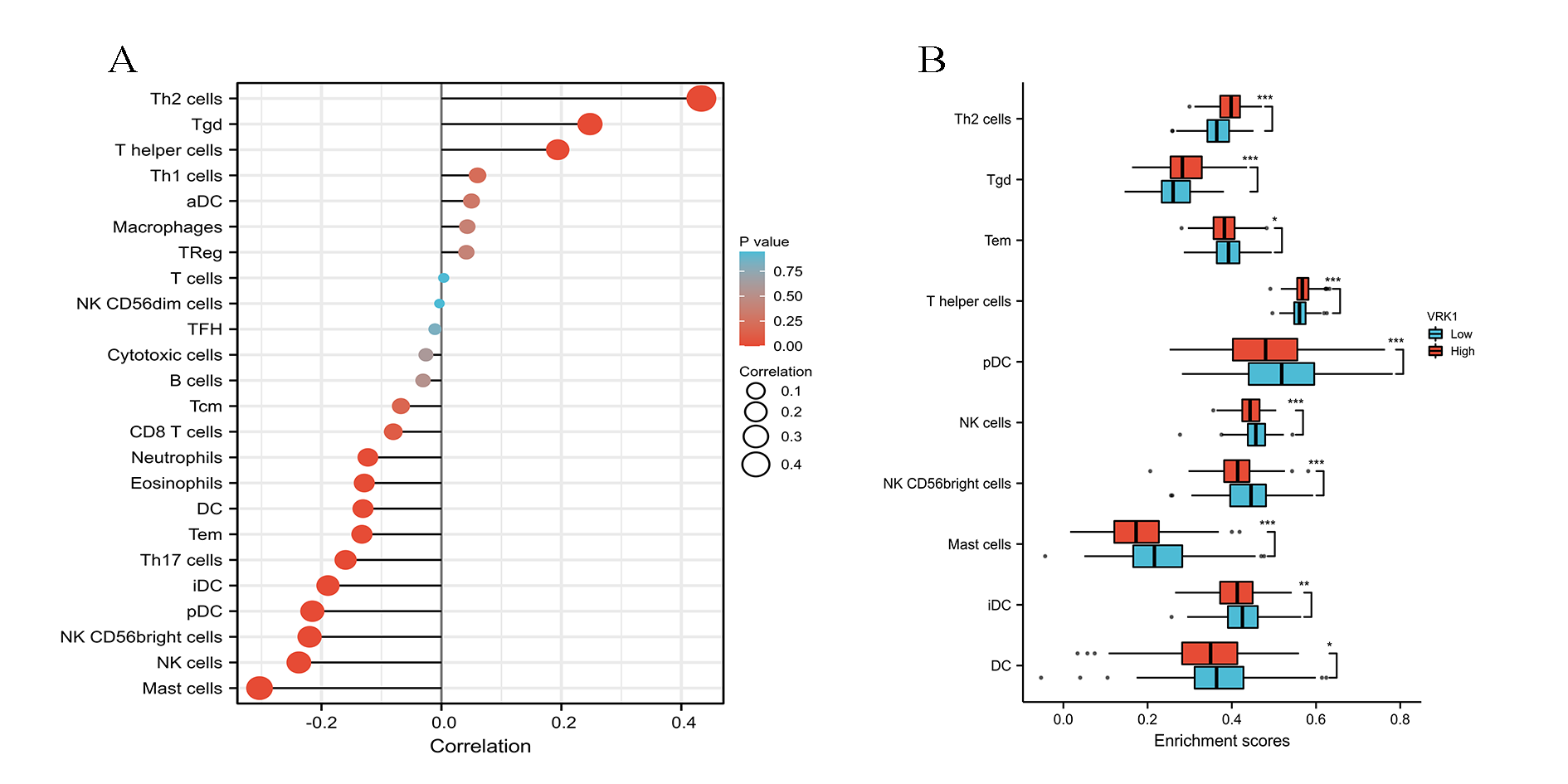

Supplement: Supplementary file 1 [file Image1.tif]

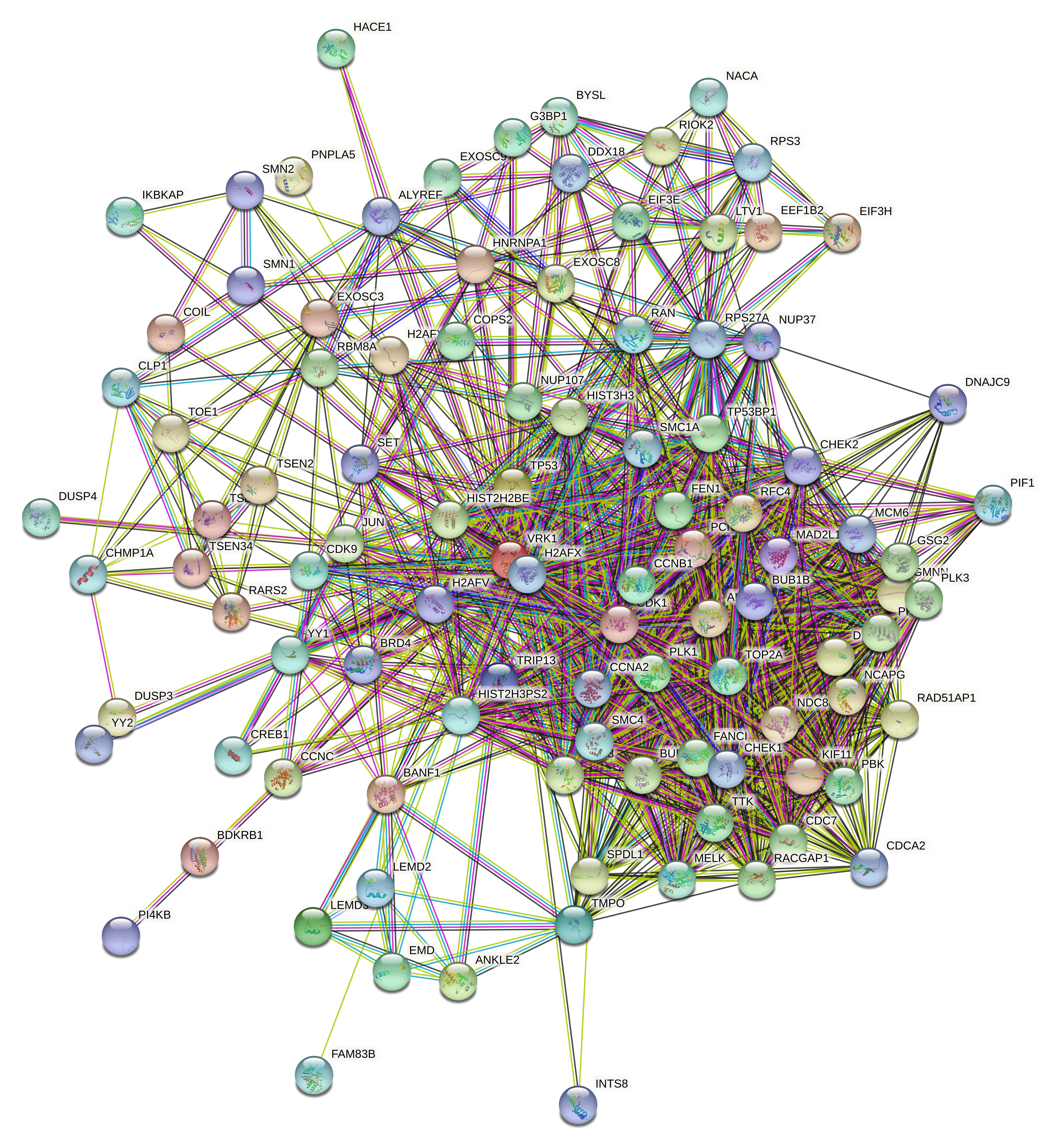

Supplement: Supplementary file 2 [file Image2.png]
